# The Clinical Utility of the MOCA in iNPH Assessment

**DOI:** 10.3389/fneur.2022.887669

**Published:** 2022-05-23

**Authors:** Eric Wesner, Lacey Etzkorn, Shivani Bakre, Jinyu Chen, Alexander Davis, Yifan Zhang, Sevil Yasar, Aruna Rao, Mark Luciano, Jiangxia Wang, Abhay Moghekar

**Affiliations:** ^1^Department of Neurology, Johns Hopkins University School of Medicine, Baltimore, MD, United States; ^2^Department of Biostatistics, Johns Hopkins University Bloomberg School of Public Health, Baltimore, MD, United States; ^3^Department of Neurosurgery, Johns Hopkins University School of Medicine, Baltimore, MD, United States

**Keywords:** cognition, normal pressure hydrocephalus, cognitive examination, Montreal Cognitive Assessment (MoCA), reliable change index (RCI)

## Abstract

**Objectives:**

We sought to estimate reliable change thresholds for the Montreal Cognitive Assessment (MoCA) for older adults with suspected Idiopathic Normal Pressure Hydrocephalus (iNPH). Furthermore, we aimed to determine the likelihood that shunted patients will demonstrate significant improvement on the MoCA, and to identify possible predictors of this improvement.

**Methods:**

Patients (*N* = 224) presenting with symptoms of iNPH were given a MoCA assessment at their first clinic visit, and also before and after tap test (TT) or extended lumbar drainage (ELD). Patients who were determined to be good candidates for shunts (*N* = 71, 31.7%) took another MoCA assessment following shunt insertion. Reliable change thresholds for MoCA were derived using baseline visit to pre-TT/ELD assessment using nine different methodologies. Baseline characteristics of patients whose post-shunt MoCA did and did not exceed the reliable change threshold were compared.

**Results:**

All nine of reliable change methods indicated that a 5-point increase in MoCA would be reliable for patients with a baseline MoCA from 16 to 22 (38.4% of patients). Furthermore, a majority of reliable change methods indicated that a 5-point increase in MoCA would be reliable for patients with a baseline MoCA from 14 to 25. Reliable change thresholds varied across methods from 4 to 7 points for patients outside of this range. 10.1% had at least a 5-point increase from baseline to post-TT/ELD. Compared to patients who did not receive a shunt, patients who received a shunt did not have lower average MoCA at baseline (*p* = 0.88) or have better improvement in MoCA scores after the tap test (*p* = 0.17). Among shunted patients, 23.4% improved by at least 5 points on the MoCA from baseline to post-shunt. Time since onset of memory problems and post-TT/ELD gait function were the only clinical factors significantly associated with having a reliable change in MoCA after shunt insertion (*p* = 0.019; *p* = 0.03, respectively).

**Conclusions:**

In patients with iNPH, clinicians could consider using a threshold of 5 points for determining whether iNPH-symptomatic patients have experienced cognitive benefits from cerebrospinal fluid drainage at an individual level. However, a reliable change cannot be detected for patients with a baseline MoCA of 26 or greater, necessitating a different cognitive assessment tool for these patients.

## Introduction

Idiopathic normal pressure hydrocephalus (iNPH) is a potentially reversible neurological condition due to altered cerebrospinal fluid dynamics, manifesting primarily in impaired gait and balance, as well as cognitive decline and urinary incontinence. iNPH is most common among older adults, however, incidence rates vary widely between studies based on the methods and operational definitions used (1). Recently, the largest study to evaluate the incidence rates of iNPH reported it to be 14.65 in 100,000 adults age 70 or older (2).

This wide range of incidence rates reflect the challenges in diagnosing iNPH. The symptomatology of iNPH has considerable overlap with more common age-related disorders, such as Alzheimer's Disease (AD). Hence, iNPH diagnosis guidelines recommend that clinicians conduct a Tap Test (TT) or extended lumbar drainage (ELD) and consider changes in their patients gait primarily when determining the likelihood of iNPH.

In addition to changes in the patient's gait, clinicians also consider improvements in their cognition when diagnosing iNPH (3, 4). Research has shown that, for some patients, cognition does significantly improve following CSF drainage either from a TT or ELD and following shunt surgery (5–9). However, these studies have focused on comparing mean differences across groups, such as gait-responders vs. non-responders (6) or shunted vs. non-shunted iNPH patients (7). Thus, clinicians still lack a validated metric for determining what is considered a “significant” cognitive improvement for individual patients. Due to the high variability of cognitive measures in impaired patients, clinicians can be misled by mild cognitive improvements (10).

Therefore, we sought to address this gap in the literature by providing an empirically-based index for determining if an iNPH patient shows significant cognitive improvement following a TT in a commonly used cognitive test, the Montreal Cognitive Assessment (MoCA). To accomplish this aim, we compared various methods for estimating reliable change indices (RCIs) for MoCA in suspected iNPH patients undergoing a TT or ELD. RCIs express change relative to their associated error which make them particularly usual for repeated cognitive measures. Thus, RCIs have emerged as a strong empirically-based approach to improving clinical decision making and have been applied to cognitive change in patients suffering from Parkinson's disease (11), strokes (12), and concussions (13).

For this study, we calculated RCIs for Montreal Cognitive Assessment (MoCA), a brief measure of cognitive ability designed to detect mild cognitive dysfunction (14). The MoCA is well validated in older adult populations (15–17), as well as populations suffering from various types of dementia (10, 18). Furthermore, the MoCA is commonly used in iNPH populations, with studies demonstrating its sensitivity to cognitive changes at several different time points following a TT (5, 6) and even surgery (19). Importantly, RCI methods have been applied to MoCA to calculate reliable change in the cognition of healthy older adults (20) and those with mild cognitive impairment (21) but not iNPH specific populations to date.

The primary aim of this study is to extend the extant literature on RCIs for the MoCA to an iNPH-symptomatic population; thereby providing clinicians with an empirically-based index for interpreting significant cognitive improvement post-TT or post-ELD. Additionally, we sought to determine whether patients with possible iNPH selected for shunt surgery based on improvements in tests of gait and balance were more likely to exhibit a reliable change in MoCA score than those who were not selected for shunt surgery. Lastly, we aimed to determine the likelihood that shunted patients will demonstrate significant improvement on the MoCA, and to identify possible predictors of this improvement so that we may better inform clinician's and patient's expectation for a significant improvement in cognition following shunt surgery at an individual level.

## Methods

### Participants

Two hundred twenty-four patients (Age: Mean = 74.4 yrs, SD = 7.8, 58% male) presenting with suspected iNPH were evaluated in the Center for CSF Disorders between October 2013 to September 2021. Patients were considered to have suspected iNPH if they presented with ventriculomegaly (Evan's Index > 0.3) and gait dysfunction with or without cognitive and urinary dysfunction, without antecedent causes. This study was approved by the Johns Hopkins IRB (Cerebrospinal Fluid Disorders Biorepository & Adult Hydrocephalus Clinical Research Network NA_00029413). All study protocols followed the guidelines set forth by the Johns Hopkins IRB. The study being a retrospective study involving only data extraction and analysis, informed consent was waived by the IRB. Data once extracted was anonymized for analysis.

### Measurements and Procedures

During the initial baseline visit, patients had their cognition assessed via the MoCA by a trained psychometrician using the standard form. Alternate forms of the MoCA were not used. Additionally, patients were administered mobility tests assessing their gait velocity [e.g., Ten Meter Walk Test (10MWT), Timed Up & Go (TUG)], balance [Mini-Balance Evaluation Systems test (Mini-BESTest)], and endurance [6-Min Walk Test (6MWT)]. Patients also completed several questionnaires, including the Neurology Quality of Life short forms on depression and executive function (NQL-D, NQL-ED), the Functional Activities Questionnaire (FAQ), and the International Consultation on Incontinence Questionnaire (ICIQ).

Furthermore, patients were asked to report the number of falls they experienced in the previous 6 months, the time since onset of their memory problems, and the time since onset of their gait problems. They also underwent an MRI or CT scan, and Evan's Index was calculated from their brain scans.

A few months after their baseline visit patients returned for one of two types of CSF drainage trial. They either received a tap test (TT) or an external lumbar drainage (ELD). During these visits, patients once again completed the MoCA and the mobility testing, both immediately before and after the procedure.

Physicians considered a number of factors to determine a diagnosis for iNPH, and patients who demonstrated a significant improvement in their gait parameters following TT/ELD were recommended shunt surgery. Patients who received a brain shunt later returned for a follow-up MoCA and gait testing. During our retrospective data extraction, we also gathered information on patients age, sex, race, level of education, and body mass index (BMI) from their medical charts.

### Statistical Analysis

Reliable changes for the MoCA were first calculated according to the nine methods outlined by Hinton-Bayre (22–31). The changes in MoCA measurements from our patients from baseline visit to pre-TT/ELD assessment constituted the data for our “normative” population. We then implemented the nine reliable change models using the changes in MoCA measurements from our patients from baseline to post-TT/ELD (22–31). We calculated the relative probabilities of exhibiting a reliable change for patients who improved following TT/ELD and were recommended shunt surgery vs. those who did not.

For all nine methods, the initial reliable change models indicated that patients with an initial MoCA score above 25 would have needed a score above 30 (the highest possible MoCA score) for an increase to be deemed reliable. Hence, in a secondary analysis, we removed participants with an initial MoCA above 25 and re-calculated the reliable change thresholds. This secondary analysis used only the four methods which did not rely on the assumption of the first and second measurements having equal variance (26–29).

To predict MoCA after insertion of a shunt for diagnosed NPH patients, we fit a series of linear regression models. First, we regressed post-shunt MoCA on each baseline, pre-TT, and post-TT MoCA separately, as well as the average of these three MoCA measurements. We selected the MoCA measurement that predicted post-shunt MoCA with the lowest adjusted R-squared for further models. We performed best subset model selection to determine which demographic characteristics (race, sex, age, education), other baseline clinical measurements (BMI, ICIQ, FAQ, NQL-D, NQL-ED, Evan's Index), and other reported information (number of recent falls, time since onset of memory and gait problems) could be used to predict MoCA after shunt insertion. We also regressed MoCA improvement on years between shunt insertion and final MoCA score measurement to understand if and how MoCA improvement may also be a function of when the score is measured after shunt insertion. All analyses were carried out using the statistical software R, version 4.0.5.

## Results

Table 1 describes the patient and clinical characteristics of the 224 patients included in our analysis. There were no significant differences in demographic characteristics (age, sex, education, race) between patients who did and did not receive a shunt. Compared to patients who did not receive a shunt, patients who received a shunt reported more falls in the 6 months preceding their first clinic visit (*p* = 0.026), took longer during the pre-TT/ELD TUG test (*p* = 0.005), and had a greater decrease in the TUG test from pre-TT/ELD to post-TT/ELD. The remaining clinical factors were not significantly different between patients who did and did not receive a shunt.

**Table 1:** Patient characteristics by diagnosis of normal pressure hydrocephalus (*n* = 224).

	**All patients**		**No shunt (*****n*** **= 153)**		**Shunt (*****n*** **= 71)**		
	**N or Median**	**(%)** **(IQR)**	**N or Median**	**(%)** **(IQR)**	**N Or Median**	**(%)** **(IQR)**	***p*** ^**1**^
Age (years)	75	(70, 80)	75	(70, 80)	74	(71, 79)	0.59
**Gender**
Female, *n* (%)	94	(42%)	62	(40.5%)	32	(45.1%)	0.62
Male, *n* (%)	130	(58%)	91	(59.5%)	39	(54.9%)	0.62
**Education**
High school, *n* (%)	72	(32.1%)	49	(32%)	23	(32.4%)	1
College, *n* (%)	82	(36.6%)	54	(35.3%)	28	(39.4%)	0.65
Graduate, *n* (%)	70	(31.2%)	50	(32.7%)	20	(28.2%)	0.6
**Race**
White, *n* (%)	202	(90.2%)	139	(90.8%)	63	(88.7%)	0.8
Black, *n* (%)	19	(8.5%)	11	(7.2%)	8	(11.3%)	0.45
Other race, *n* (%)	3	(1.3%)	3	(2%)	0	(0%)	0.55
BMI (kg/m^2^)^a^	27.1	(24.5, 30.7)	26.6	(24.3, 30.7)	27.7	(25.3, 30.7)	0.49
Evan's index (x100)	36	(33.8, 40)	36	(33, 39)	37	(35, 40)	0.06
Number of falls in past six months (count)^b^	2	(0, 4)	2	(0, 4)	3	(1, 5)	0.026*
**Time since onset of**
Gait problems (months)^c^	18	(9, 36)	18	(9.5, 36)	18.5	(9.8, 36)	0.85
Memory problems (months)^d^	12	(4, 24)	12	(4, 25)	12	(6, 24)	0.57
**MoCA**
Baseline	22	(18, 25)	22	(18, 25)	22	(19, 24)	0.88
Pre-TT/ELD	22	(18, 24)	21	(17, 24)	22	(18, 24)	1
Post-TT/ELD	22	(18, 25)	22	(18, 25)	22	(18, 26)	0.41
**MoCA improvement**
Baseline to pre-TT/ELD	0	(-3, 2)	0	(-3, 2)	0	(-3, 2)	0.59
Baseline to post-TT/ELD	1	(-2, 3)	0	(-2, 2)	1	(-1, 3)	0.17
Pre to post-TT/ELD	1	(-1, 3)	1	(-1, 3)	1	(0, 3)	0.31
**Timed Up/Go**,^**a**^
Pre-TT/ELD (s)	16	(11.7, 24.8)	15.1	(11.4, 22.3)	18.4	(12.9, 37.9)	0.005*
Post-TT/ELD (s)	13.1	(10.3, 21.3)	12.7	(10.2, 20.1)	14.9	(10.6, 26.2)	0.24
Improvement (s)	−1.8	(-4.6,−0.4)	−1.2	(-2.7,−0.1)	−4.6	(-10.8,−1.8)	<0.001*

1 *P-Values were generated using a Wilcoxon test for continuous variables and a Fisher's exact test or chi-square test for categorical variables. a. Missing value in 1 patient; b. Missing values in 16 patients; c. Missing values in 3 patients; d. Missing values in 7 patients*.

* *P-Value < 0.05*.

**Figure 1:**
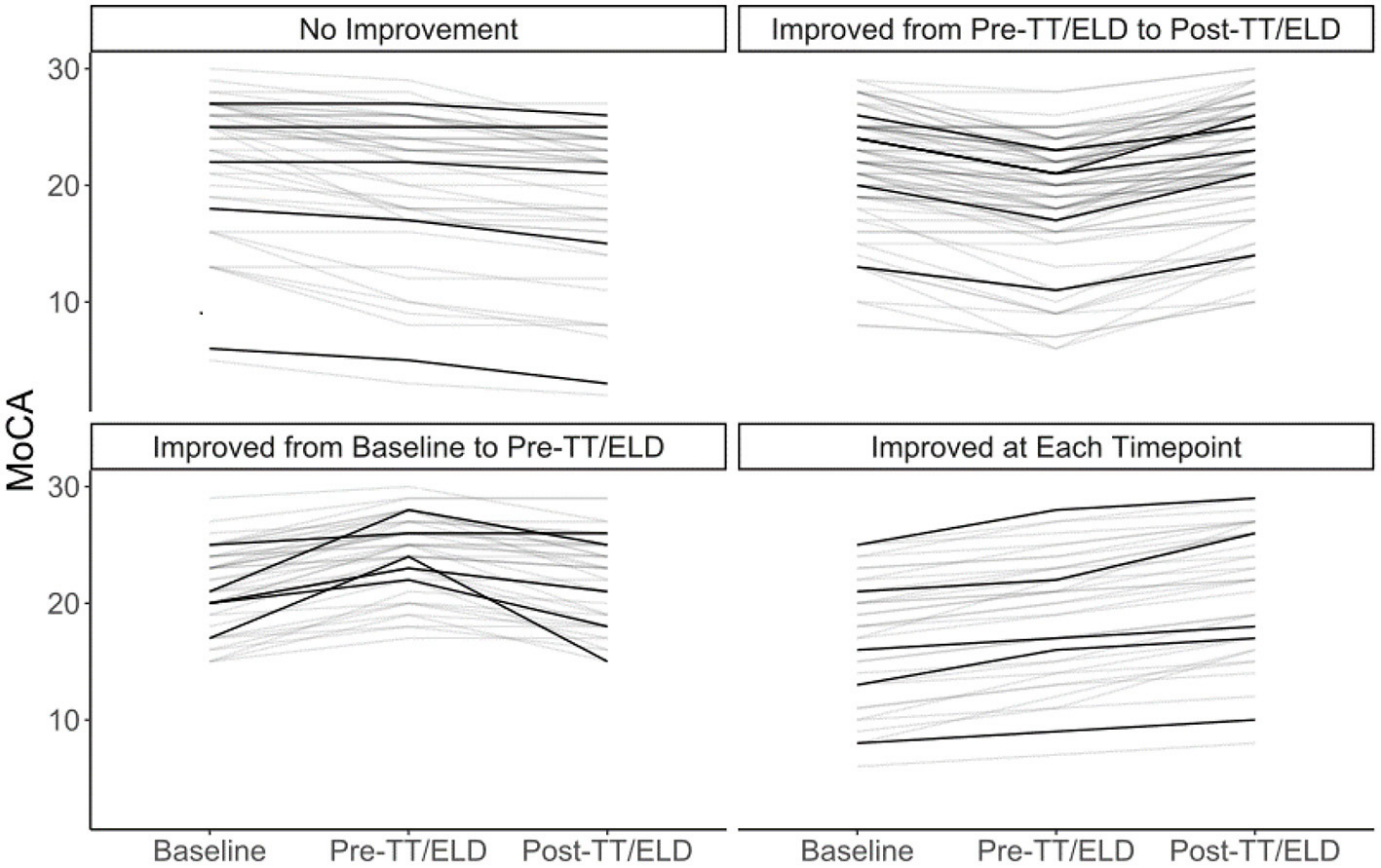
Individual trajectories of MoCA at baseline, pre-TT/ELD, post-TT/ELD. 5 representative patients in each group identified in darker lines. No improvement (*n* = 49). Improvement from pre to post-TT/ELD (*n* = 90). Improvement from baseline to pre-TT/ELD (*n* = 47). Improvement from baseline to pre-TT/ELD and pre to post-TT/ELD (*n* = 38).

In terms of change of MoCA scores between baseline and before and after LP/ELD's, four distinct groups could be observed (Figure 1). Forty Nine patients showed no improvement, 90 showed an improvement from pre to post-TT/ELD, 47 showed an improvement from baseline to pre-TT/ELD and finally 38 showed an improvement from baseline to pre-TT/ELD & pre to post-TT/ELD. Across the whole group, there was no significance between the MoCA score at pre-TT/ELD [median (IQR): 22 (18, 24)] and the MoCA score at baseline [median (IQR): 22 (18, 25)] for patients.

**Figure 2:**
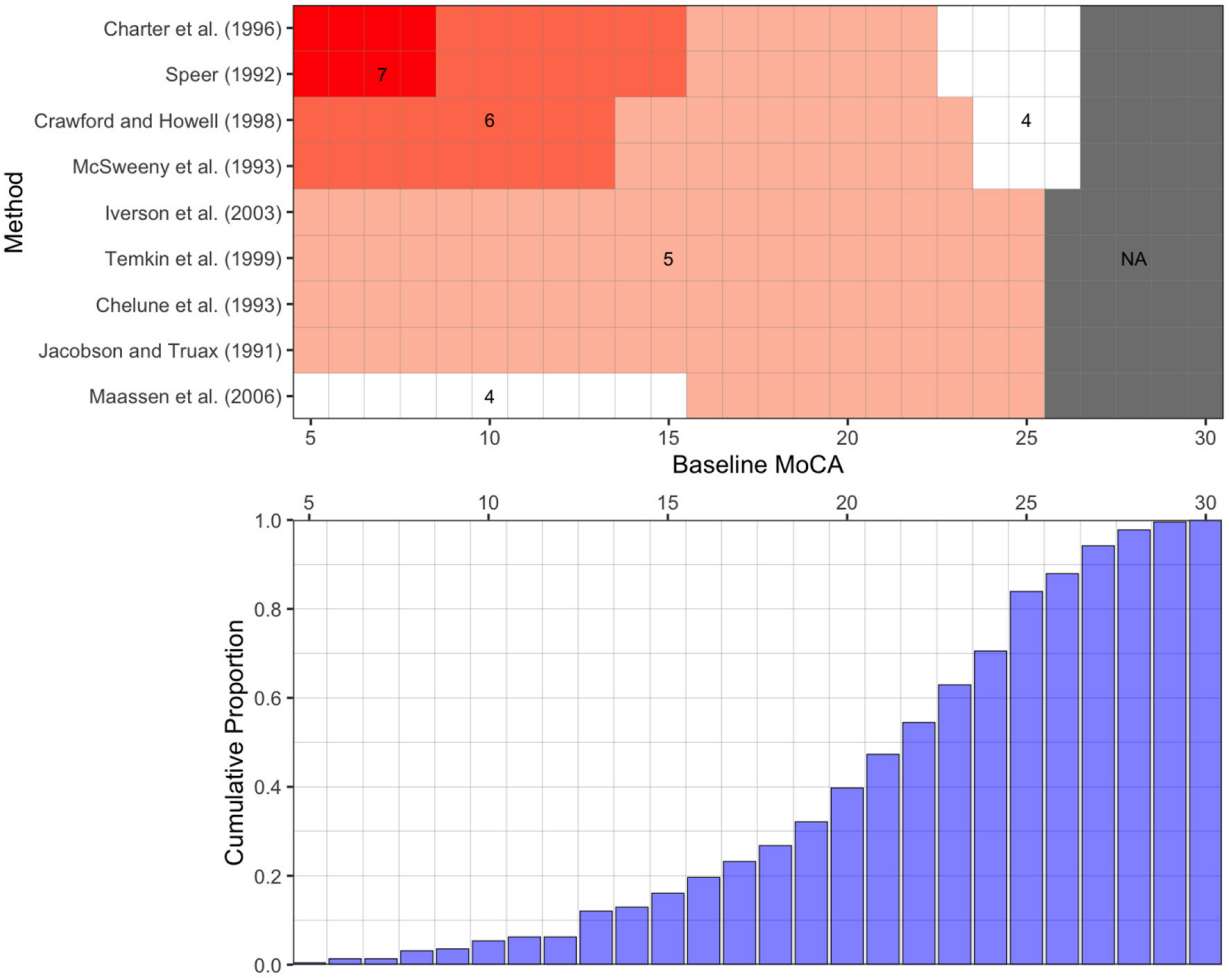
Thresholds for Reliable Increases for the Montreal Cognitive Assessment among Patients with Suspected Normal Pressure Hydrocephalus using nine Methods. Top Panel: Given an initial MoCA score, the minimum reliable increase for a change in MoCA for nine methods. Reliable increases were generated using the 95th percentile of the prediction distribution (z = 1.64). Across all methods considered, a minimum increase of 5 points could be deemed reliable for a patient who initially presented with a score of 20. No reliable change could be determined for patients presenting with a score above 25, as the maximum possible MoCA score is 30. Bottom Panel: The proportion of patients presenting at or below a given MoCA score at baseline. For example, 20% of patients had a score of 16 or less at baseline.

**Figure 3:**
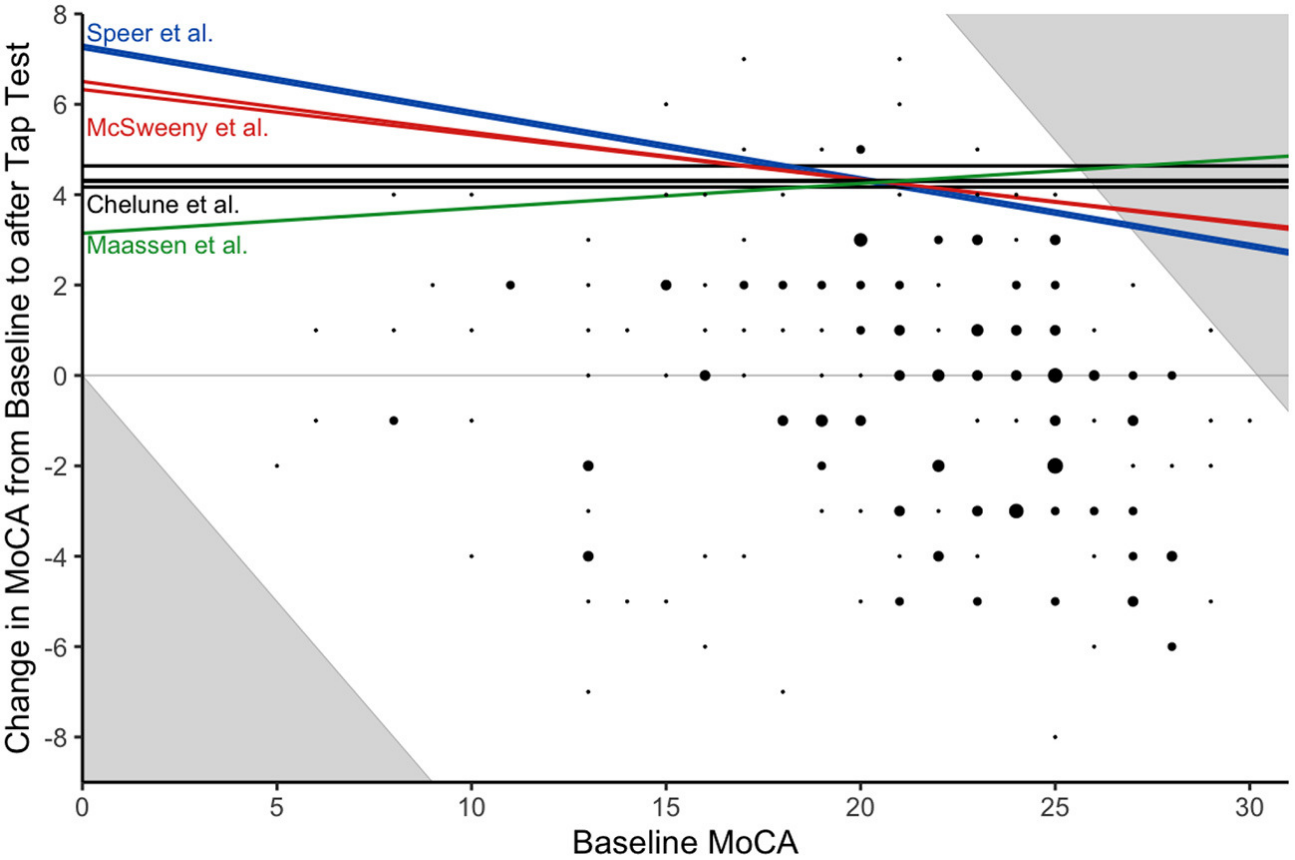
Comparison of Changes in the Montreal Cognitive Assessment to Multiple Reliable Increase Thresholds. Thresholds for all nine reliable change methods are overlaid on the data. The choice of slope formula for the reliable change threshold was more consequential than the choice of intercept formula. Hence, each reliable change threshold is given the color corresponding to the slope formula used (i.e., Speer, McSweeny, Chelune, or Maassen et al.). Since MoCA is a discrete measurement and overlapping points were common in the scatterplot, the size of the point indicates the number of participants with that particular (x, y) combination.

Figure 2 depicts the calculated reliable change thresholds using nine methods. All methods agreed that a 5-point increase in MoCA would be reliable for patients with a baseline MoCA from 16 to 22 (38.4% of patients). Reliable change thresholds varied from 4 to 7 points for patients outside of this range. The threshold varied based on the method used as well as the baseline MOCA score. Figure 3 illustrates the nine thresholds against a scatter plot of baseline MoCA scores by changes in MoCA from baseline to post-TT/ELD.

Table 2 shows percent of patients considered having a reliable change after the TT/ELD for patients who did and did not receive a shunt. The values were roughly similar across those groups. The percent of patients with a reliable change of 5 or more points for those who got a shunt ranged from 23 to 28% depending on the method used.

**Table 2:** Percent patients labeled as having a reliable change in MoCA from baseline to tap test, shunt insertion.

	**No shunt,** **Post-TT/ELD** **(***n* **= 129)**^***a***^	**Shunt,** **Post-TT/ELD** **(***n* **= 59)**^***a***^	**Shunt,** **Post-shunt** **(*****n*** **= 47)**^***a***^
Jacobson and Truax (23)	10.1	10.2	23.4
Speer (24)	10.1	11.9	27.7
Chelune et al. (25)	10.1	10.2	23.4
McSweeny et al. (26)	10.9	10.2	25.5
Charter et al. (27)	10.1	11.9	27.7
Crawford and Howell (28)	10.9	10.2	25.5
Temkin et al. (32)	10.1	10.2	23.4
Iverson et al. (30)	10.1	10.2	23.4
Maassen et al. (31)	13.2	13.6	25.5

a *Patients with baseline MoCA of 25 or lower*.

The best predictor of MoCA after shunt insertion was the average MoCA score prior to shunt insertion (adjusted R-Squared = 0.65, Table 3). Further model selection efforts revealed that after accounting for the average prior MoCA measurement, no further demographic or clinical factors helped to predict post-shunt MoCA. Table 3 also shows, that while average of pre-shunt MoCA's is the best predictor, the other MoCA scores (baseline MoCA, pre-TT/ELD MoCA, and post-TT/ELD MoCA) are still good predictors of post-shunt MoCA (Adjusted R-Squared = 0.56, 0.57, 0.58, respectively).

**Table 3:** Univariate regressions of post-shunt MoCA on prior MoCA measurements.

**Measurement**	**Intercept** ^***a***^	**(SE)**	**Slope**	**(SE)**	**Adjusted** **R-Squared**
Baseline MoCA	22.46	(0.44)	0.70	(0.08)	0.56
Pre-TT/ELD MoCA	22.24	(0.43)	0.74	(0.09)	0.57
Post-TT/ELD MoCA	21.99	(0.43)	0.76	(0.09)	0.58
Average pre-shunt MoCA	22.23	(0.39)	0.84	(0.08)	0.65

a *MoCA measurement were centered at their respective mean values given in Table 1*.

Table 4 compares the characteristics of patients with vs. without reliable MoCA change (≥5) from baseline to post-shunt. There are no significant differences in most of these characteristics except for time since memory problems, which is shorter for patients who displayed a reliable change, and post-TT/ELD TUG, which is worse for patients who display a reliable change after shunt surgery. This is mainly a function of the different pre-TT/ELD TUG times. Subjects that improved on the MoCA by 5 or more had worse pre-TT/ELD TUG times (30.7 vs. 18.3 s) which was nearly significant (*p* = 0.06). The subjects with improvement in MoCA 5 or more did demonstrate a trend in greater improvement in TUG [-9.6 (95%CI:−15.6,-5.7) vs.−4.8 (95%CI:−11,-2.6), *p* = 0.16] following TT/ELD.

**Table 4:** Patient characteristics by change in MoCA from baseline to post-shunt (*n* = 56)^*a*^.

	**MoCA Improvement**				
	** <5** **(*****n*** **= 36)**		**≥5+** **(*****n*** **= 11)**		***p*** **-value** ^***b***^
Age (years)	75.5	(71, 79.2)	74	(72.5, 78.5)	0.92
**Sex**
Female, *n* (%)	14	(38.9%)	4	(36.4%)	1
Male, *n* (%)	22	(61.1%)	7	(63.6%)	1
**Education**
High school, *n* (%)	15	(41.7%)	5	(45.5%)	1
College, *n* (%)	14	(38.9%)	4	(36.4%)	1
Graduate, *n* (%)	7	(19.4%)	2	(18.2%)	1
**Race**
White, *n* (%)	31	(86.1%)	9	(81.8%)	0.66
Non-white, *n* (%)	5	(13.9%)	2	(18.2%)	0.66
BMI (kg/m^2^)	27.2	(25.7, 30.5)	29.6	(27.8, 31.7)	0.2
Evan's INDEX (x100)	37	(36, 40)	38	(33, 39.5)	0.5
Falls in past 6 months (count)	3	(1, 6)	2	(0.2, 5.5)	0.74
Time since onset of gait problems (months)	19	(12, 36)	18	(11.5, 30)	0.85
Memory problems (months)	12	(6.5, 36)	8	(0, 12)	0.019*
Timed up/Go Pre-TT/ELD (s)	18.3	(14.6, 39.2)	30.7	(21.5, 55.9)	0.06
Post-TT/ELD (s)	14.8	(10.9, 25.3)	26.8	(16, 40.3)	0.03*
Change (s)	−4.8	(−11, −2.6)	−9.6	(−15.6, −5.7)	0.16

a *Excludes patients with baseline MoCA > 25. ^b^P-Values were generated using a Wilcoxon test for continuous variables and median (IQR) were presented for continuous variables and a Fisher's exact test for binary variables*.

* *P-Value < 0.05*.

**Figure 4:**
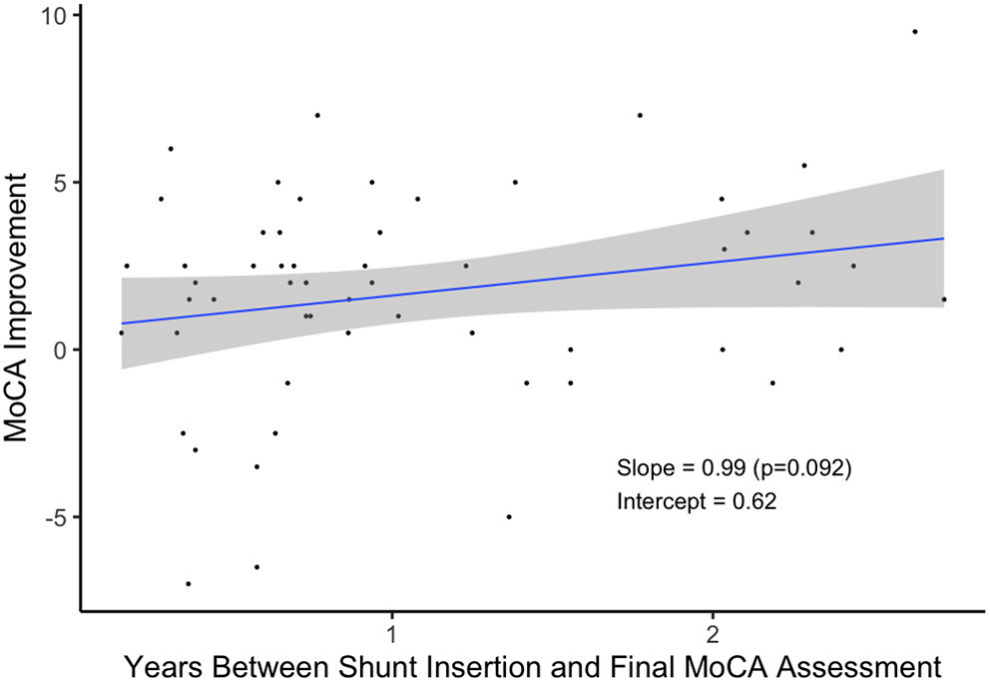
Improvement in MoCA by Time Since Shunt Insertion (*n* = 56).

Patients who received a shunt and waited longer to take their final MoCA assessment tended to exhibit greater improvement in their scores (Figure 4). On average, patients scored nearly one point higher for each additional year they waited, but more data is needed to verify this relationship (*p* = 0.092).

Supplementary Table 1 provides more detailed information on the equations used to calculate reliable changes. Supplementary Figures 1, 2 detail the reliable change models fit only using patients with baseline MoCA of 25 or lower.

## Discussion

The MoCA has recently become a widely used and standard screening tool for cognitive function, including in patients with iNPH. However, the thresholds used to define improvement in an iNPH population are not well defined and only group comparisons have been described. The present study used multiple methods to estimate reliable change thresholds for the MoCA in a population of older adults with suspected iNPH who underwent a CSF drainage procedure (TT/ELD) and a subset of them underwent shunt surgeryIf validated, this reliable change threshold could serve as a guide to patients and clinicians in management of iNPH patients.

While the Mini Mental Status Examination [MMSE; (33)] is one of the most widely used cognitive screening tests, the MoCA is now considered to be a more suitable and sensitive test for the evaluation of cognition in patients with Mild Cognitive Impairment or early dementia (34, 35). Such cognitive screening instruments are also used repeatedly to assess the progression of cognitive disorders or the response to a specific intervention. To be able to distinguish between true or reliable changes vs. changes occurring as a result of measurement error or chance, reliable change indices have been developed both for the MMSE (36) and MoCA in different populations (20). In the latter population-based study of 197 cognitively normal healthy older adults (age: 60–94 yrs) followed longitudinally for 4 years, the reliable change index for the MoCA was 4. In another population-based study of 128 cognitively normal and mildly impaired participants in whom the MoCA was administered twice with an interval of 2–4 months, the 95% minimal detectable change for the MoCA was 4 (16). This study closely mimics our patient population both in terms of age and the time interval between MoCA administrations, including the lack of use of alternate versions of the MoCA.

Prior research on the MoCA demonstrates that this brief measure is not only valid in iNPH populations, but sensitive to changes in cognition following TT (5, 6). As a result, the MoCA was selected as a tool to measure and monitor cognition in the Adult Hydrocephalus Clinical Research Network as well as in a prospective trial of shunt surgery for iNPH (19, 37, 38). In contrast to such studies evaluating group-level differences, our findings provide clinicians with an index for determining if CSF drainage has resulted in a reliable change in MoCA scores at a patient specific level with respect to their baseline MoCA.

Our analyses show that clinicians should look for a 5-point increase in MoCA as an indication that an iNPH-symptomatic patient has benefited cognitively from CSF drainage, 1 point higher than what has been reported for cognitively normal individuals. For patients that scored lower than 14 points on the MoCA, the methods give more mixed results, although most still calculated RCIs of 5–6 points. Finally, while several methods provided RCIs of 4 points for patients scoring a 26 on the MoCA, most of the methods indicated that a reliable change could not be detected for patients who presented with a MoCA score of 26 or greater, as the maximum possible MoCA score is 30. Therefore, a different and more comprehensive cognitive assessment may be needed to assess changes in cognition for suspected iNPH patients with higher baseline cognitive function.

Among the various clinical factors that we examined (e.g., Evan's Index, number of falls, etc.), the only ones that demonstrated a significant association with post-shunt cognitive improvement was the time since onset of memory problems and whether their gait improves. The shorter the duration of memory symptoms, the higher the likelihood of a reliable improvement in the MoCA score. Not surprisingly, a reliable improvement in MoCA was also more likely in those whose gait improved significantly. This association with respect to gait improvement replicates the findings of Matsuoka et al. (6), however, their study had a relatively small (*N* = 32) and homogenous (100% Japanese) sample. Hence, we have bolstered the generalizability of these findings by replicating them in a significantly larger and more racially/ethnically diverse sample.

There are, however, several caveats and limitations to these findings that one should consider. First, the majority of suspected iNPH patients undergoing a TT/ELD do not exhibit significant improvements in cognition as measured by the MoCA as opposed to delayed improvement after shunt surgery. This is also to be expected as TT/ELD are part of the selection process for shunt surgery and thus a significant percentage of patients undergoing a TT/ELD are typically not selected for shunt surgery at our institution. Among patients who received a shunt, a higher percentage (25%) did demonstrate an improvement in MoCA after shunt insertion compared to their performance at baseline vs. the improvement immediately after a TT/ELD (Table 2). However, there was marginally significant evidence (*p* = 0.092) that patients who waited longer to perform the final MoCA assessment improved a greater amount. Hence, improvement in cognition following insertion of a shunt may be delayed as compared to improvement in gait, balance, and urinary continence for iNPH patients. In this study patients after shunt surgery were followed for a mean of 1.10 yrs (SD: 0.72). Longer follow-up assessments are needed to determine if these improvements are sustained.

Another caveat of applying methods for calculating reliable change criteria, is that there remains a debate in the field as to which method is best. Currently, there is no clear best method for calculating reliable change criteria (31), so we chose to compare nine of the leading methods in our study. While all of the methods agreed that an improvement of 5 points was a reliable change for patients who scored 16–22 prior to CSF drainage, the methods were conflicted on RCIs for patients who scored outside of this range. A majority of the methods still calculated an RCI of 5 for scores as low as 14 and as high as 25 and this would apply to a majority of patients with iNPH presenting at specialist centers. Lastly, we did not use alternate forms of the MoCA at baseline and pre-TT/ELD that could have accounted for practice effects (39) since that is not our routine practice in clinic. However, our analysis did not demonstrate such practice effects in this large sample as has been reported in another study in which MoCA was administered in a community sample of cognitively normal and MCI participants (21).

In conclusion, this study is the first to provide practical, empirical standards which clinicians can use when assessing potential cognitive improvement following CSF drainage. These findings can be used to improve the clinical decision making of clinicians assessing iNPH at a patient level in contrast to group differences assessed in reported research studies. As a rule of thumb, a 5-point increase in MoCA scores is indicative of a reliable cognitive improvement for iNPH-symptomatic patients following CSF drainage. Furthermore, clinicians should factor in the time since onset of patient's memory problems and whether their gait improves, when advising patients about the likelihood of a significant and reliable cognitive improvement following shunt surgery. Additional studies are needed to increase the generalizability of these findings. Particularly, future research should seek to replicate and extend these findings in populations with more diverse racial and educational backgrounds. Finally, researchers should use longer follow-up intervals to determine if symptom improvements are maintained.

## Data Availability Statement

The raw data supporting the conclusions of this article will be made available by the authors, without undue reservation.

## Ethics Statement

The studies involving human participants were reviewed and approved by Johns Hopkins IRB (Cerebrospinal Fluid Disorders Biorepository & Adult Hydrocephalus Clinical Research Network NA_00029413). The patients/participants provided their written informed consent to participate in this study.

## Author Contributions

EW: conducted literature review, extracted data from medical records, and drafting/revision of the manuscript. LE: drafting/revision of the manuscript for content and including medical writing for content. SB and JC: data analysis and interpretation of results. AD, AR, and ML: major role in the acquisition of data. SY: major role in the acquisition of data and drafting/revision of the manuscript for content. JW: revised the manuscript for content and data analysis and interpretation of results. AM: study design, revision of the manuscript, major role in the acquisition of data, and interpretation of results. All authors read and approved the final manuscript.

## Funding

We received philanthropic funding from the Lantry Family Foundation.

## Conflict of Interest

The authors declare that the research was conducted in the absence of any commercial or financial relationships that could be construed as a potential conflict of interest.

## Publisher's Note

All claims expressed in this article are solely those of the authors and do not necessarily represent those of their affiliated organizations, or those of the publisher, the editors and the reviewers. Any product that may be evaluated in this article, or claim that may be made by its manufacturer, is not guaranteed or endorsed by the publisher.
