# Supplementary material for: The Clinical Utility of the MOCA in iNPH Assessment
Source: Front Neurol. 2022 May 23;13:887669. doi: 10.3389/fneur.2022.887669 (PMC9168991; doi:10.3389/fneur.2022.887669)
Supplement: Supplementary file 1 [file Table_1.docx]

**Supplementary Table 1:** Reliable Change Equations

**Table A:** Coefficients for Score Prediction in Multiple Reliable Change Methods

|  | Intercept | Slope |
| --- | --- | --- |
| Chelune et al. (1993) | -0.47 | 1.00 |
| Speer (1992) | 2.60 | 0.85 |
| McSweeny et al. (1993) | 0.90 | 1.62 |
| Maassen et al. (2006) | -1.62 | 1.06 |

**Table B:** Estimates for Standard Deviation in Multiple Reliable Change Methods

|  | Estimate |
| --- | --- |
| Christensen and Mendoza (1986) | 2.91 |
| Jacobson and Truax (1991) | 2.82 |
| Maassen et al. (2004) | 2.90 |
| McSweeny et al. (1993) | 2.86 |
