# Supplementary figures and images for: The Clinical Utility of the MOCA in iNPH Assessment
Source: Front Neurol. 2022 May 23;13:887669. doi: 10.3389/fneur.2022.887669 (PMC9168991; doi:10.3389/fneur.2022.887669)

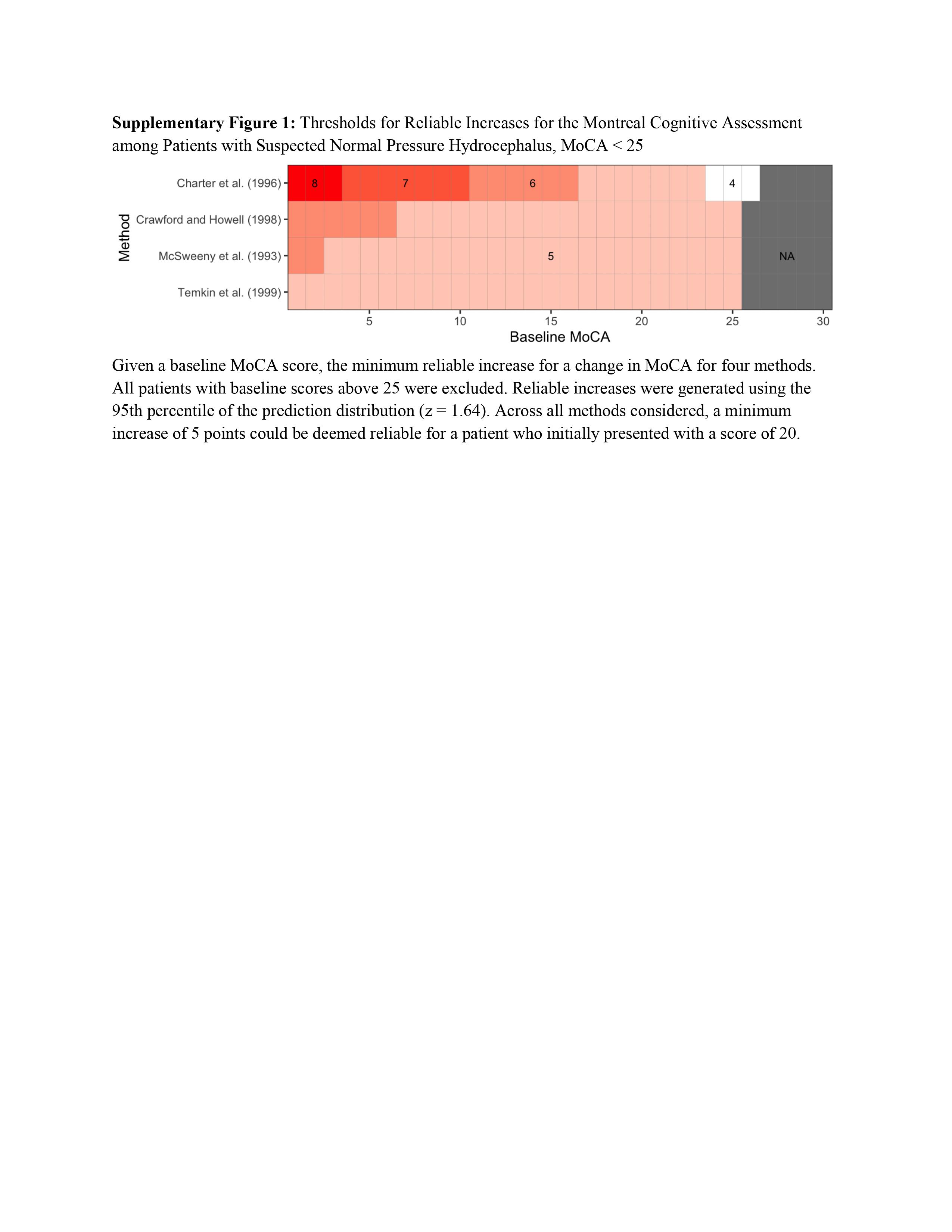

Supplement: Supplementary file 2 [file Image_1.jpg]

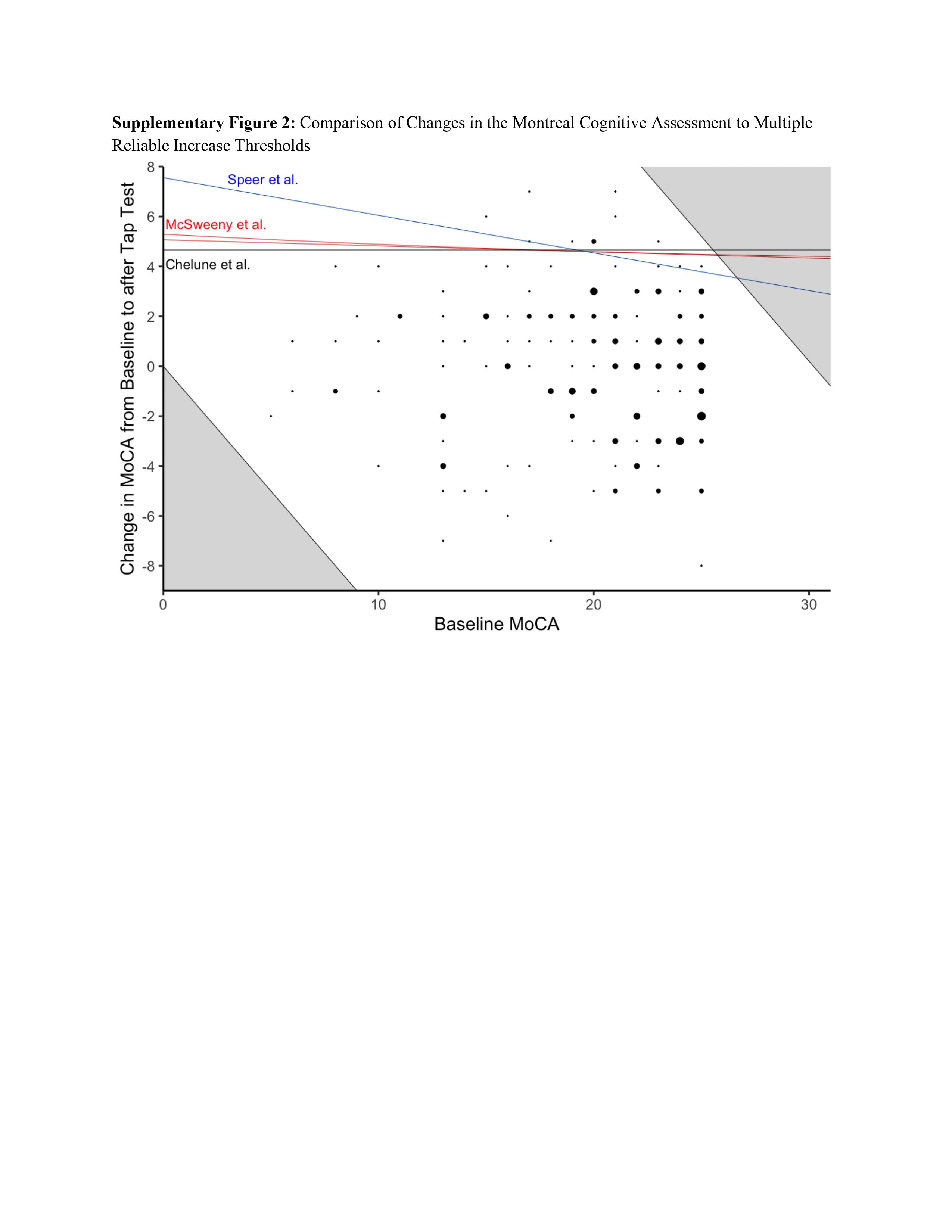

Supplement: Supplementary file 3 [file Image_2.jpg]
